# A Selection of Platforms to Evaluate Surface Adhesion and Biofilm Formation in Controlled Hydrodynamic Conditions

**DOI:** 10.3390/microorganisms9091993

**Published:** 2021-09-21

**Authors:** Luciana C. Gomes, Filipe J. M. Mergulhão

**Affiliations:** LEPABE—Laboratory for Process Engineering, Environment, Biotechnology and Energy, Faculty of Engineering, University of Porto, Rua Dr. Roberto Frias, 4200-465 Porto, Portugal; luciana.gomes@fe.up.pt

**Keywords:** biofilm, microbial adhesion, hydrodynamics, shear stress, shear rate, computational fluid dynamics, flow systems, microplates

## Abstract

The early colonization of surfaces and subsequent biofilm development have severe impacts in environmental, industrial, and biomedical settings since they entail high costs and health risks. To develop more effective biofilm control strategies, there is a need to obtain laboratory biofilms that resemble those found in natural or man-made settings. Since microbial adhesion and biofilm formation are strongly affected by hydrodynamics, the knowledge of flow characteristics in different marine, food processing, and medical device locations is essential. Once the hydrodynamic conditions are known, platforms for cell adhesion and biofilm formation should be selected and operated, in order to obtain reproducible biofilms that mimic those found in target scenarios. This review focuses on the most widely used platforms that enable the study of initial microbial adhesion and biofilm formation under controlled hydrodynamic conditions—modified Robbins devices, flow chambers, rotating biofilm devices, microplates, and microfluidic devices—and where numerical simulations have been used to define relevant flow characteristics, namely the shear stress and shear rate.

## 1. Introduction

Biofilms are surface-attached communities of microorganisms, establishing three-dimensional structures composed of bacteria surrounded by a self-made matrix [1]. This matrix consists of polysaccharides, proteins, and extracellular DNA and influences biofilm structure and morphology [2]. It is estimated that more than 90% of the bacterial cells in natural environments reside in a biofilm [3], since it gives protection against hostile conditions (pH changes, lack of nutrients, hydrodynamics, and antimicrobial compounds), encourages gene transfer, and facilitates the colonization of niches [4].

The established model for biofilm development includes five steps, starting with the (i) reversible attachment of cells to a preconditioned surface, (ii) production of extracellular polymeric substances (EPS) causing irreversible cell attachment, (iii) early development of biofilm architecture, (iv) biofilm maturation, and (v) cell dispersion from the biofilm into the surrounding environment [5,6]. An immersed substratum is rapidly covered by molecules from the liquid, forming a conditioning film that may change the properties of that surface, making it more or less suitable for bacterial adhesion [7,8]. Then, cell adsorption at the surface occurs, followed by release or reversible adhesion. The physical forces associated with conditioning film formation and reversible adhesion are electrostatic and van der Waals forces, as well as hydrophobic interactions [9]. The next step starts when the cells become irreversibly attached to the surface due to the presence of stronger attractive forces, such as covalent and hydrogen bonds, and cellular surface structures, such as fimbriae and flagella [9]. After maturation, biofilm growth and detachment/sloughing balance each other so that the biomass amount is approximately constant in time, i.e., the steady-state is attained.

Biofilm development is a problem faced by the environmental, industrial, and biomedical areas. Regardless of the setting where it takes place, it is well known that biofilm establishment and growth are affected by different factors, such as surface properties, nutrient availability, hydrodynamics, temperature, pH, and microbial cell properties [10]. Among these factors, hydrodynamics will be considered in detail in this review.

In the environment, biofilms particularly affect the efficiency of shipping, aquaculture, and coastal industries [11]. The fouling phenomenon increases the surface roughness of the ship hulls, hence increasing the friction between the fouled hull and the water [12]. This resistance increases fuel consumption and, consequently, the emissions of greenhouse gases to the atmosphere, while reducing the maneuverability of the vessel [11,12]. Additional problems related to biofouling in the environment are associated with immersed offshore structures (cages, netting, and pontoons), onshore equipment, and structures such as pumps, pipelines, and filters, due to the high drag and accelerated biocorrosion to which they are exposed [13].

Besides affecting cleaning and disinfection, biofilms formed in industrial facilities can reduce energy transfer in heat exchangers, obstruct fluid flow, and cause localized corrosion attacks [14]. It has been reported that biofilm development in industries corresponds to approximately 30% of the plant operating costs [15]. In the case of the food industry, biofilms have a detrimental effect not only on the process but also on the final product or consumer. The Center for Disease Control and Prevention (CDC) has estimated that between 1996 and 2010, 48 million illnesses, 128,000 hospitalizations, and 3,000 deaths occurred annually in the US due to the dissemination of foodborne pathogens [16].

In the biomedical field, the sessile cells are responsible for infections, as they are usually more resistant to antimicrobial therapy than their planktonic counterparts and less susceptible to host defenses [17]. The National Institutes of Health (NIH) revealed that 65% of all microbial infections are caused by biofilms [18], which can grow in indwelling medical devices and have an estimated direct cost burden of 7 billion EUR in Europe alone [19]. Biofilms formed in medical devices may contain pathogenic organisms and cause changes in surface properties and material degradation, affecting the functionality of the medical setting [20].

The ubiquitous nature of biofilms and their increasing resistance impose great challenges for the use of conventional antimicrobials and suggest the need for combined or multi-targeted approaches. In this sense, the study of strategies capable of preventing microbial adhesion through the modification of surface properties (for instance, making them antimicrobial and/or antiadhesive) may be one of the simplest and most reasonable ways to inhibit surface colonization and delay biofilm growth [21,22,23,24]. However, few studies have evaluated the effectiveness of these promising surfaces in conditions that mimic real scenarios, particularly regarding hydrodynamics. In this review, the most commonly used platforms for the in vitro assessment of microbial adhesion and biofilm formation under flow conditions—modified Robbins devices, flow chambers, rotating biofilm devices, microplates, and microfluidic devices—are introduced, and their main advantages and disadvantages are discussed. These testing platforms have been used transversally in the environmental, industrial, and medical fields, mainly with the aim of evaluating the effects of different substratum features, microbial strains, and shear forces on adhesion and biofilm formation, due to their ability to control the hydrodynamics (flow rate and/or shear stress or shear rate) and recreate in vivo flow conditions. This becomes a critical step in translating research into practical applications.

## 2. Effects of Hydrodynamics on Microbial Adhesion and Biofilm Formation

The flow conditions of each system where there is a surface material (natural, industrial, or biomedical) have a very strong influence on the biofilm onset. During initial adhesion, hydrodynamics dictates the rate at which macromolecules (specific for each type of fluid) and microorganisms are delivered to the surface, the time they reside close to the surface, and the shear forces at the surface-fluid interface [25]. According to Katsikogianni and Missirlis [26], there is an optimum flow rate for bacterial adhesion, reflecting the balance between the rate of cell delivery and the force acting on adhered bacteria. Furthermore, the bacteria–substratum interaction determines the shear forces that adhered bacteria will be able to withstand [26].

Besides the relevant role of hydrodynamics on the microbial adhesion step, it is also one of the most important factors in biofilm formation and structure. The fluid surrounding a biofilm is the source for nutrients and vehicle for cell by-product removal [27]. An increase in flow velocity promotes the flux of molecules (nutrients, cells, biocides, antibiotics, metabolites, etc.) by changing their concentrations in the biofilm–fluid interface. Hydrodynamics also regulates the physiological properties of the biofilm by changing the mechanical shear stresses at the interface [25]. Higher shear forces often lead to the formation of thinner, denser, and stronger biofilms [28]. Although higher flow velocities enhance molecular transport by convection, the higher density of biofilms reduces the diffusivity of the molecules inside them [29,30]. Additionally, stronger shear forces can be responsible for higher biofilm sloughing or detachment [28].

Given the importance of shear forces on initial adhesion and biofilm development, it is essential to characterize them. The vast majority of biofilm studies under flow conditions only report the tested flow rate. Nevertheless, the flow rate by itself provides little information about shear forces since it does not take into consideration the geometry of the flow system. Two main parameters should be considered to characterize shear effects: the shear rate and the shear stress. Mathematically, the shear rate is the derivative of the velocity in the perpendicular direction from the wall system [31] and quantifies the frequency at which cells contact the surface. The shear stress in Newtonian fluids is proportional to the shear rate, where fluid viscosity is the constant of proportionality [31], translating the friction from the fluid acting on the adhered cells or the biofilm. Therefore, shear stress is commonly used as a descriptor of the shear forces acting on the biofilm during maturation or detachment.

Computational fluid dynamics (CFD) are commonly used to model biofilm reactors because they enable the estimation of the fluid flow parameters of these systems, such as the shear stress and the shear rate, at relatively low cost and faster, in comparison to experimental techniques [32,33]. CFD requires that the geometry to be analyzed is divided into a finite set of volumes, called cells, forming a computational grid, called mesh. Fluid flows are described by differential equations for the conservation of mass, momentum, and energy; CFD replaces these equations with algebraic equations, which can be numerically solved for each cell, resulting in a flow field [34]. These equations describe how the single operating parameters are related. Although CFD is very useful for understanding biofilm behavior, one must bear in mind that most simulations are performed for clean surfaces. When biofilms are formed, the cross-sectional flow area is reduced, increasing the bulk flow velocity and wall shear stress. Thus, these simulations are particularly recommended for the study of initial adhesion, early stages of biofilm development (such as those usually investigated in biomedical settings), and surfaces that are frequently cleaned (as is the case with food processing equipment). In these situations, the thickness of the formed biofilms is unlikely to have a significant impact on flow dynamics and shear forces distribution [35].

## 3. Biofilm Platforms

In this context, biofilm reactors are platforms for the study of biofilms in laboratory conditions. One of the major obstacles to study in vitro biofilms is the choice of a suitable platform, where key variables such as flow rate and shear stress can be manipulated in order to mimic the conditions found in real scenarios. Although completely reproducible biofilms are nearly impossible to obtain, the development of in vitro platforms for biofilm studies is a foremost step towards the standardization of procedures and for better control of the environmental conditions that affect biofilm development [36,37].

Here, we describe the most commonly used platforms for microbial adhesion and biofilm formation in controlled hydrodynamic conditions, particularly those where CFD has been used to determine relevant flow characteristics. These platforms have advantages and limitations, which are summarized in Table 1.

### 3.1. Flow Cells: Robbins Device and Modifications, and Flow Chambers

Flow cells can be generally divided into two types: those that are based on the design of the Robbins device and those that are built for the direct inspection of biofilm development, here called flow chambers. In both types of flow cells, it is possible to test different surface materials simultaneously in similar nutritional and hydrodynamic conditions. Nevertheless, it is worth mentioning that modified Robbin devices have higher throughput and hydrodynamic range than flow chambers. The Robbins device and its modifications present a higher number of sampling ports available for analysis, allowing for multiple biofilm samples to be taken simultaneously, as well as for sampling more than a single time point during biofilm development [39]. Although both types of flow cells are useful tools for studying biofilm under controlled conditions, they need a specialized apparatus, are technically challenging, and are not suitable for rapid high throughput assays. Another weakness of these systems is that only a single microbial strain can be analysed per experiment.

The most straightforward configuration of a flow cell system is that of a bioreactor containing a batch culture of the desired microorganism so that the content of the reactor is pumped through the flow cell and the effluent drained to waste. This configuration may be interesting for adhesion studies, particularly if the flow rates to be tested are low, since the duration of the assay is limited by the cell suspension volume. Another configuration is to place the flow cell in a recycle loop so that the culture volume is no longer a limitation and assays can last longer and perform at higher flow rates [40]. However, it has the disadvantage that the composition of the batch culture is always changing. A third alternative is to have a chemostat feeding a recirculation loop so that the feed flow to the chemostat equals the drain flow from the loop. In this case, it is possible to feed the flow cell with a constant concentration of cells and nutrients, while decoupling the flow rate going through the flow cell from the dilution rate [41,42]. With this flow cell configuration, it is possible to work at very high flow rates and attain high shear stresses that are comparable to those found in the environment and industry [43].

#### 3.1.1. Robbins Device and Modifications

The Robbins device was initially developed by Jim Robbins and Bill McCoy to monitor biofilm formation in industrial water systems [44]. Several modifications were later introduced to this design, including the use of a square-channel pipe where coupons are aligned with the inner surface without disturbing flow characteristics [45]. They are convenient for studies where a large biofilm mass amount is wanted. With the modified Robbins devices, the flow can be momentarily stopped to allow direct access to the coupon, so that time-course experiments are possible. This stop of the flow system for coupon removal involves some risk because, even if the operator is very careful that the shutdown and restart of the system are smooth, there may be some loosening of the biofilm already formed in the remaining coupons of the flow cell. For quantitative analysis of the biofilm to be carried out, destructive sampling techniques are usually required. Conventional techniques, such as total and viable cell counts, as well as protein and carbohydrate content analysis, comprise the disruption of the biofilm [42,46].

Other flow cell designs include a half-pipe geometry that more closely resembles the geometry of piping systems [43,47]. These flow cells can be operated either in laminar or turbulent regimes, but it is important to guarantee that the flow cell has an entry section that is long enough to allow for flow development before the sampling zone (thus avoiding entry effects) and that the effect of the sudden contraction on the exit zone is negligible. This will ensure that all coupons are subjected to the same hydrodynamic conditions and that biofilm samples can be directly compared [48].

**Figure 1:**
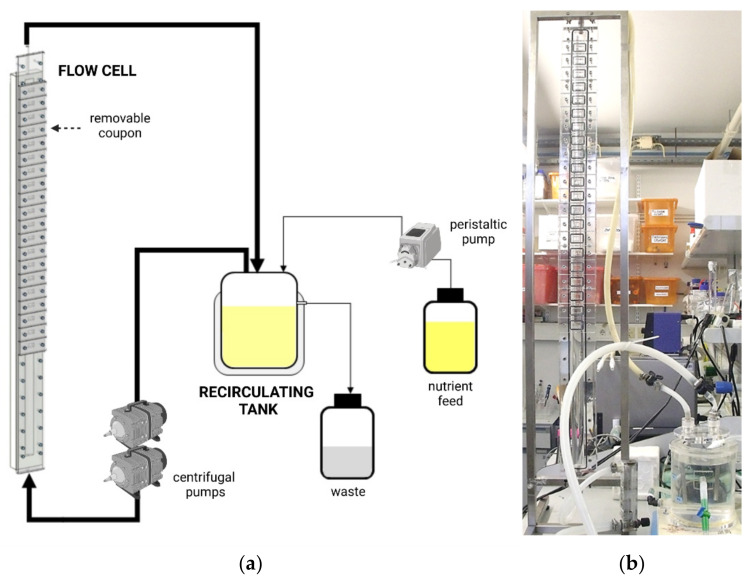
Modified Robbins device with the fluid behavior fully characterized by CFD: (**a**) schematic representation and (**b**) photograph. The system is mainly composed of a recirculating tank and one vertical semi-circular flow cell (about a meter high) with removable coupons, as well as peristaltic and centrifugal pumps.

In our group, a custom-made, semi-circular flow cell (identical to that shown in Figure 1) was designed to evaluate the performance of different surface coatings in preventing biofouling in the marine environment [22], food industry [24,41], and medical devices [49,50]. The hydrodynamics of this flow cell system was fully characterized by CFD [48], which allows not only the guarantee that all sampling coupons are exposed to the same shear forces but also provides knowledge of the flow rate and Reynolds number, which is necessary in order to operate this platform and simulate the shear stress and/or shear strain described for different real scenarios.

#### 3.1.2. Flow Chambers

In spite of the many advantages of modified Robbins devices, they are neither adequate for monitoring the initial cell adhesion to a surface nor for the direct analysis of biofilm development. For these purposes, several models of flow chambers that can be mounted on a microscope stage and used with video capture systems have been developed, enabling real-time observation of microbial adhesion, particularly when used with transparent surfaces. The employment of fluorescent probes coupled with confocal laser scanning microscopy (CLSM) makes flow chambers especially appreciated for in situ gene expression studies [51].

The most well-known flow system to study cell adhesion is the parallel-plate flow chamber (PPFC) developed by Bos et al. [52]. Adhesion can be studied in the PPFC system under controlled hydrodynamics that mimics, for instance, physiologically relevant conditions [40,53] using a wide range of microorganisms and surfaces with different properties. This system requires low volumes and, consequently, has a reduced cost when compared to modified Robbins devices; additionally, it presents one or more glass viewing ports that permit non-destructive, real-time adhesion (single-cell visualization) and biofilm observation. Despite their versatility, one must bear in mind that PPFCs have a much lower throughput than microplates and larger flow cells based on the Robbins device. Additionally, when real-time monitoring of adhesion is performed, a decrease in the initial adhesion rates is often observed along the experimental time, which is related to a phenomenon called hydrodynamic blocking [54,55]. Hydrodynamic blocking can reduce the adhesion of cells since the area behind each adhered cell is screened from incoming cells. Adhesion rates obtained in such conditions are not truly representative of the interaction between a single cell and the surface. Thus, initial adhesion assays in these setups should be conducted so that low surface coverage is attained, and the absence of blocking should be confirmed so that consistent results can be obtained [54].

**Figure 2:**
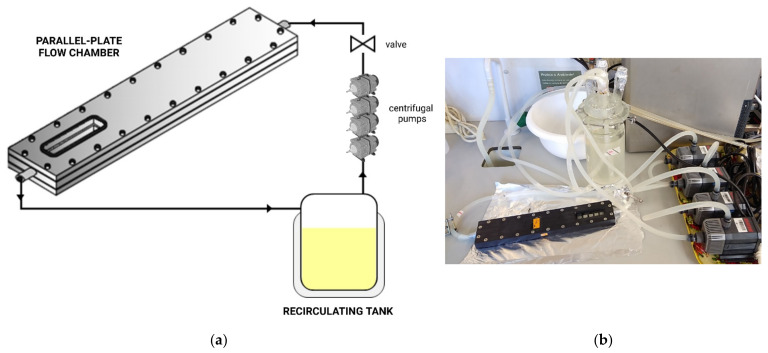
Flow chamber system setup: (**a**) schematic representation and (**b**) photograph. The PPFC is coupled to a glass tank connected to four centrifugal pumps and a tubing system to conduct adhesion or biofilm formation assays.

Flow chamber systems have been designed to analyse cell adhesion [23,56,57] and biofilm formation [58,59], including a PPFC coupled to a jacketed tank and connected to centrifugal pumps and a valve via a silicone tubing system (Figure 2). The valve allows the bacterial suspension to circulate through the system at a controlled flow rate [40], and the recirculating water bath is connected to the tank jacket to enable temperature control.

### 3.2. Rotating Biofilm Devices

**Figure 3:**
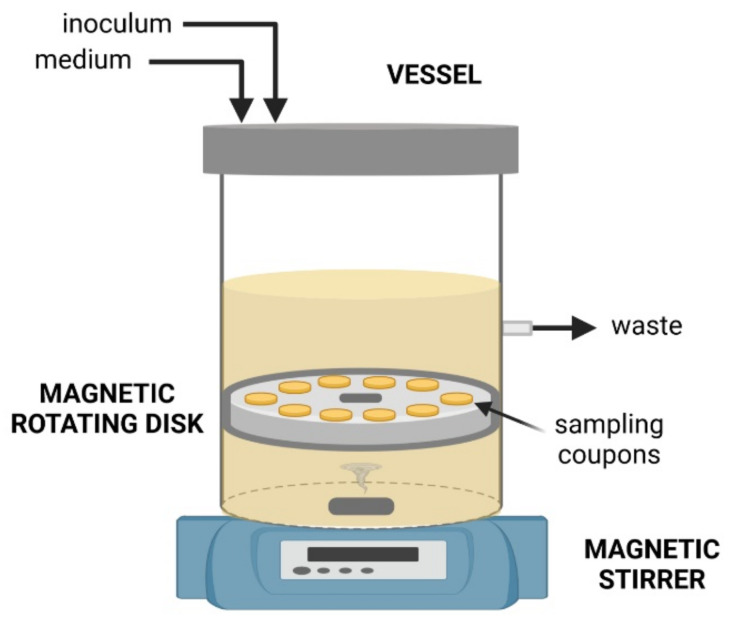
Scheme of a rotating disk reactor.

Two types of rotating biofilm reactors are commonly used in the assessment of material and fluid flow effects on biofilm development: the rotating disk reactor and the rotating cylinder reactor. These reactors have different designs. The rotating disk reactor consists of a 1-L vessel with a magnetically driven rotor at the bottom, which holds removable coupons for biofilm formation (Figure 3) [60]. The hydrodynamic conditions under which the biofilm is formed are controlled by adjusting the disk rotation speed [60], and the shear stress on the coupons’ surface can be estimated from the Navier–Stokes equations. The rotating cylinder reactor is often composed of four cylindrical sections that can be rotated at variable speeds within four concentric chambers [61]. Unlike the rotating disk reactor, this platform can be used to test different cell suspensions, since each chamber of the cylinder reactor has independent feeding and sampling ports [61].

### 3.3. Microfluidic Devices

**Figure 4:**
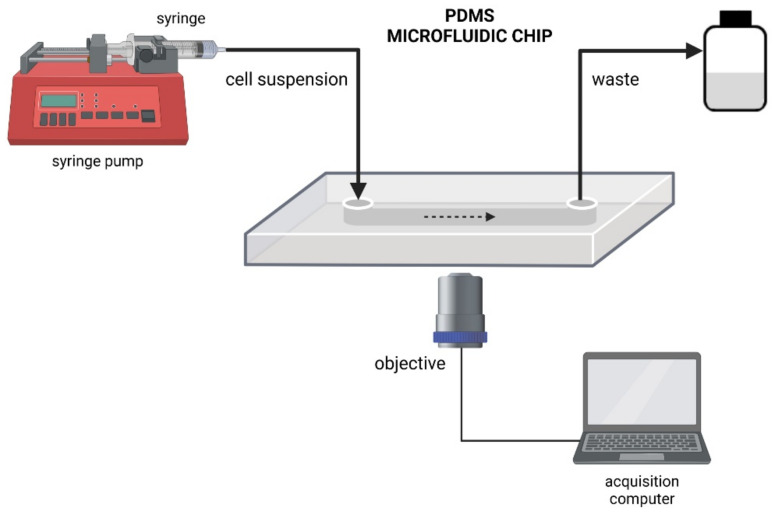
Schematic diagram of a microfluidic setup.

Microfluidic platforms have demonstrated high potential and versatility for the study of microbial adhesion and biofilm formation under different growth conditions. Compared with traditional flow cell systems, microfluidics enables greater control over flow conditions, can be used to explore a much wider range of shear rates with high flexibility in designing different flow geometries, and facilitate the parallelization of experiments [62,63]. Although microfluidic devices can be fabricated by different techniques and from a diversity of materials, the flexible elastomer polydimethylsiloxane (PDMS) has been the material of choice for the construction of these devices. Several other surfaces can be studied using xerographic construction techniques that enable different polymers to be incorporated into microfluidic flow cells [53]. Concerning the analysis methods, although off-chip detection with conventional methods is feasible, on-chip detection by optical and/or fluorescence microscopy is preferred, in order to visualize in situ and real-time effects (Figure 4) [36].

Although there is a tendency to develop biofilm models in miniaturized devices, microfluidic-based devices also have their limitations: the small liquid volumes used in microfluidics may further impede molecular analysis, and the spatial confinement may generate different biofilms from those formed in more open systems [64]. Additionally, this platform requires specialized technical abilities for device fabrication and experimental setup, and system clogging can occur due to the small dimensions [36]. Air bubbles are another recurring issue in microfluidics [65]. Because of the micrometric dimensions of the tubes and channels, air bubbles can be very difficult to remove, leading to fluid flow instability and most likely to the detachment of adhered cells or biofilm portions.

### 3.4. Microplates

Microplates are currently the most widely used platform for biofilm development studies. They consist of plates with multiple wells arranged in a rectangular array with a 2:3 aspect ratio, resulting in 6, 12, 24, 48, 96, and 384 wells. The volume of each well can range from tens of microliters to few milliliters, depending on the number of wells [66]. Although most researchers use microplates in static conditions, they can be placed in orbital incubators and used for dynamic biofilm studies under controlled fluid conditions [67,68]. These devices are easy to handle, which allows for studying the adhesion of different microbial strains and consequent biofilm formation in rapid and inexpensive assays, due to their reduced volume [69]. Depending on the format used, they enable high throughput at an affordable cost and sometimes non-invasive imaging through optical coherence tomography (OCT) [70,71] and confocal laser scanning microscopy (CLSM) [72]. Particularly for larger well dimensions, it is possible to place coupons at the bottom of the wells so that different surface materials can be tested [70,73,74]. The main limitations of microplates are that loosely attached cells may not be measured correctly due to detachment during washing and that biofilms formed in this platform are affected by sedimentation.

#### 3.4.1. 96-Well Microplates

This is the most intensively used microplate format, mainly for screening purposes. Biofilm formation in this platform is severely affected by sedimentation, and the direct inspection of the biofilm is possible but technically difficult [75,76]. They are particularly suited for short-term experiments, as they operate in batch mode with the intrinsic exhaustion of nutrients and accumulation of toxic metabolites. Results obtained in this platform often lack reproducibility, possibly due to the washing steps that are researcher-dependent and the existence of several protocol versions for biofilm analysis [36]. These plates are generally not compatible with the use of coupons, as the bottom surface is relatively small; so, only a limited number of surfaces can be assayed (limited to the construction materials of these plates). 

#### 3.4.2. 12- and 6-Well Microplates

These microplates are very attractive formats. Although theoretically their throughput is lower than the 96-well plates, the results obtained with these platforms are more reproducible due to the higher liquid volume, decreasing the need for a large number of replicate wells. These two types of plates also sustain microbial growth for longer periods, but medium replacement can be necessary. Large coupons can be used for biofilm formation (square surfaces of up to 1.5 cm can be placed on the bottom of the 12-well plates), and uniform shear forces can be obtained. Even though the shear stress in the coupon varies with the radial distance to the center, each coupon has identical average shear stress values [71].

**Figure 5:**
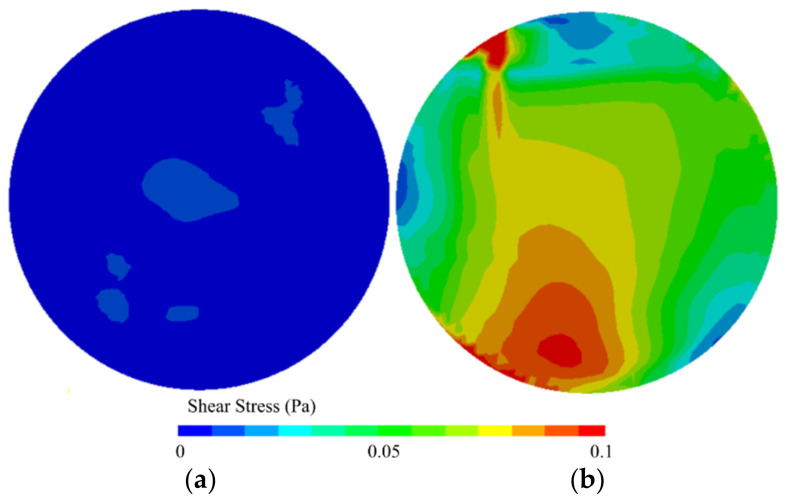
Shear stress magnitudes (Pa) on the bottom of 12-well microplates shaken at (**a**) 40 and (**b**) 180 rpm (orbital diameter of 25 mm, liquid volume of 3 mL, and temperature of 25 °C). Adapted from Gomes et al. [78].

The hydrodynamics inside the wells of 12-well microplates have been simulated to assess the effect of orbital shaking frequency on shear stress. Numerical simulations were performed at 25 °C, with an orbital diameter of 25 mm, a liquid volume of 3 mL, and shaking frequencies of 40 and 180 rpm (Figure 5). As expected, higher shear stresses at the bottom of the wells can be attained at higher shaking frequencies; values up to 0.07 Pa and shear rates of 42 s^−1^ were achieved. These values are much higher than those obtained with 96-well microplates [8,77]. 

## 4. Adhesion and Biofilm Studies Performed under Controlled Hydrodynamics

In this section, illustrative examples of the application of the described in vitro platforms are given, when appropriate, for the investigation of initial microbial adhesion, biofilm formation and its treatment under controlled shear conditions in different fields: environment, industry, and medicine.

### 4.1. Environmental Applications

Table 2 presents typical shear values that can be found in the environmental field. In a natural environment, a shear rate range between 4 and 125,000 s^−1^ can be obtained.

Most of the research in this area has been devoted to the impact of shear and surface characteristics on biofilm formation, giving less relevance to microbial cell adhesion (Table 3). It was also observed that flow systems, namely modified Robbins devices and rotating biofilm devices, are the main choice to emulate the turbulent flows and high wall shear stresses found in water systems [79,80,81]. However, in the last few years, efforts have been made to predict flow conditions in easy-to-handle biofilm platforms like microplates [68,71]. A detailed hydrodynamic analysis of the 12-well microplates [71] allows us to define the operational conditions that should be used in the laboratory bench to further assess the biofilm formation capacity of marine bacteria [70,71] and the antibiofilm activity of novel surface coatings [22,82] under hydrodynamic conditions prevailing in natural aquatic environments.

**Table 2:** Environmental scenarios and their typical shear ranges.

Environmental Scenario	Shear Stress (Pa)	Shear Strain (s^−1^)	References
Drinking-water distribution systems	0.13–9.10	n.d.	[79,83]
Ship in harbor	n.d.	50	[84]
Ship navigation (turbulent flow)	n.d.	125,000	[84]
Marine environments	n.d.	4 and 40	[71]
Tumbling and pouring	n.d.	10–100	[84]
Channels within a biofilm	n.d.	60–300	[84]

n.d.—not disclosed.

### 4.2. Industrial Applications

Similar to what was observed in environmental systems (Table 3), in the industrial field, the modified Robbins devices and rotating devices were the most reported reactors for biofilm formation and treatment studies. Different groups have used these flow systems in shear stress intervals of great amplitude [94,95,96], covering a huge range of shear values that can be found in the industry (Table 4). Our research group, in particular, has operated a semi-circular flow cell system (Figure 1) in different conditions and was able to attain shear stress values up to 0.6 Pa during biofilm formation [42,48], confirming the versatility of this platform and its capacity to mimic the hydrodynamic conditions that can be found, for instance, in the food industry (Table 4).

When the aim was to study microbial adhesion in an industrial environment, biofilm researchers preferred to use flow chambers [97,98] or microplates [24,41], since they are faster to operate and may allow for direct inspection by microscopic techniques (Table 5).

**Table 4:** Examples of industrial processes and their associated shear stress ranges.

Industrial Equipment or Phenomenon	Shear Stress (Pa)	References
Pipeline elbows	0.009	[99]
Dead ends	0.05–18.9	[100]
Removal of deposits from stainless steel tubes	0.09	[101]
Corners of a washing tank	<0.1	[102]
Angles of a washing tank	0.1–0.4	[102]
Mix proof valve	0–0.25	[103]
Three-way valve	0.4–1.7	[104]
Half-open butterfly valve	0–190	[100]
Product fill valve	0–1180	[105]
Milk spray dryer	0–0.4	[106]
Cleaning-in-place pilot plant	0–5	[107]
Plate heat exchanger for yoghurt processing	6.7 and 20–46	[108,109]
Plate heat exchanger of an ice slurry system	50–100	[110]
Pilot-scale plate heat exchanger for milk treatment	150–450	[108]

### 4.3. Biomedical Applications

Several studies were found in the literature where biofilm assays were performed under characterized hydrodynamic conditions similar to those of medical settings. Depending on the biomedical scenario, the shear stress range can vary between 0.02 and 88.3 Pa, and the shear strain between 0.1 and 80,000 s^−1^ (Table 6). Flow chambers have particularly been used in the medical field to evaluate the antiadhesive activity of novel surface materials for biomedical devices, including urinary tract and implanted devices (Table 7), since they are adequate for low fluid shear stresses and laminar flow applications, as well as for real-time insight into the dynamic process of microbial cell adhesion [21,40,57]. Furthermore, the dimensions of the flow cell or the flow rate can be adjusted to attain the required shear stress/shear rate, in order to resemble in vivo flow conditions.

Microfluidic platforms have also demonstrated high potential and flexibility for the study of microbial adhesion [113,114] and biofilm formation [115,116] under different hydrodynamic conditions.

**Table 6:** Characteristic shear conditions found in biomedical scenarios.

Human Body or Biomedical Device	Shear Stress (Pa)	Shear Strain (s^−1^)	References
Blood flow in veins	0.076–3.4	20–800	[117,118]
Blood flow in arteries	0.2–1	50–650	[117,118]
Fluid in the oral cavity	n.d.	0.1–50	[84]
Kidney collecting duct cells	0.02–2	n.d.	[119]
Uterus	<0.1	n.d.	[120]
Cerebral circulation	n.d.	>100	[121]
Urinary catheter	n.d.	15	[84,122]
Hemodialysis catheter	52.6–88.3	20,000–80,000	[123]
Catheter sheath introducer	0.03	n.d.	[124]
Endovascular stent	0.22–6.72	n.d.	[125]
Prosthetic valve	0.06–27.84	n.d.	[126]
Contact lens motion	n.d.	1000	[127]

n.d.—not disclosed.

**Table 7:** Biomedical studies performed on different biofilm platforms to evaluate the initial adhesion, as well as the biofilm formation and treatment under the defined shear conditions.

**Platform**	**Field**	**Biofilm Stage**	**Study Aim**	**Hydrodynamics**	**Assay Time**	**Surface Material**	**Organisms**	**Concluding Remarks**	**References**
**Modified Robbins device**	General medical devices	Biofilm formation	Effect of flow rate variation on mass transfer and biofilm development	Flow rates of 374 and 242 L h^−1^, corresponding to shear stresses between 0.183 and 0.511 Pa	9 days	Polyvinyl chloride	*Escherichia coli*	Biofilm formation was favored at the lowest flow rate because shear stress effects were more important than mass transfer limitations. This flow cell system generates wall shear stresses that are similar to those found in some biomedical settings.	[111]
	Urinary devices	Biofilm formation	Evaluation of the potential of antiadhesive coatings when immobilized onto medical-grade polyurethane	Flow rate of 53 mL s^−1^, corresponding to 15 s^−1^	48 h	Polyurethane Polyurethane coated with CyanoCoating through a polydopamine layer application, or O_2_- plasma, N_2_-plasma, and O_3_ activation	*Escherichia coli*	When the coating was produced via O_3_ activation, CyanoCoating was able to decrease the biofilm biovolume by 88% and the surface coverage by 95%, compared to the uncoated surface.	[50]
			Investigation of the role of uncommon bacteria on the *Escherichia coli* microbial consortium	Flow rate of 300 mL min^−1^, corresponding to 15 s^−1^	72 h	Silicone rubber	*Escherichia coli* *Delftia tsuruhatensis*	*E. coli* and *D. tsuruhatensis* were able to form single- and dual-species biofilms. Both bacteria tend to co-aggregate and cooperate over time, persisting in a stable microbial community.	[128]
			Development of new functional coatings using magnetron co-sputtering to deposit triple TiO_2_/SiO_2_/Ag nanocomposite thin films	Flow rate of 53 mL s^−1^, corresponding to 15 s^−1^	48 h	Glass TiO_2_/SiO_2_ coated glass with different Ag contents (0 to 19.8 at %)	*Escherichia coli*	Biofilm formation was reduced down to 92% compared to a control glass surface. The coatings are promising candidates for antimicrobial protection of urinary tract devices for at least 48 h, suggesting benefits over longer periods.	[49]
**Flow chamber**	General medical devices	Adhesion	Assessment of interactions of bacteria with specific biomaterial surface chemistries under flow conditions	50, 500, 1000, and 2000 s^−1^	2 h	Glass Glass with alkyl silane monolayers	*Staphylococcus epidermidis*	The increase in the ionic strength enhanced adhesion to the different surfaces, in accordance with the Derjaguin–Landau–Verwey–Overbeek (DLVO) theory, under low shear rates. The increase in the shear rate restricted the predictability of the theory.	[129]
			Effect of shear stress on bacterial adhesion to biomedical materials	Flow rates of 2 and 4 mL s^−1^, corresponding to shear stresses of 0.01 and 0.022 Pa	0.5 h	Glass Polydimethylsiloxane Poly(L-lactic acid)	*Escherichia coli*	Similar adhesion rates were obtained on glass and polydimethylsiloxane. The highest adhesion rates were obtained on glass and polydimethylsiloxane, and the lowest on poly(L-lactic acid).	[40,53]
			Effect of fluid composition and shear conditions on bacterial adhesion to an antifouling peptide-coated surface	Flow rates of 2 and 4 mL s^−1^, corresponding to 15 and 30 s^−1^	0.5 h	Glass Peptide-coated glass Poly(L-lactic acid)	*Escherichia coli*	Adhesion reductions of 40–50% were attained at a shear rate of 15 s^−1^ on the peptide-coated surfaces compared with glass. The performance of the peptide-based antifouling coating was superior to poly(L-lactic acid).	[57]
			Effect of shear stress on bacterial adhesion to antifouling polymer brushes	Flow rates of 2 and 4 mL s^−1^, corresponding to 0.010 and 0.024 Pa	0.5 h	Glass Poly[N-(2-hydroxypropyl) methacrylamide] brush Poly[oligo(ethyleneglycol) methyl ether methacrylate] brush	*Escherichia coli*	Both polymer brushes reduced the initial adhesion up to 90% when compared to glass.	[56]
			Evaluate the antiadhesive activity of carbon nanotube composites	Flow rate of 2 mL s^−1^, corresponding to 15 s^−1^	0.5 h	Polydimethylsiloxane Carbon nanotube/polydimethylsiloxane composites	*Escherichia coli*	The introduction of carbon nanotubes composites in the polydimethylsiloxane matrix yielded less bacterial adhesion than the polydimethylsiloxane alone. Less adhesion was obtained on the composites with pristine rather than functionalized carbon nanotubes. Incorporation of higher amounts of carbon nanotubes in polymer composites can affect bacterial adhesion by more than 40%. Composites enabling a 60% reduction in cell adhesion were obtained by carbon nanotube treatment by ball-milling.	[23,130]
	Devices and implants	Adhesion	Prevention of microbial adhesion to silicone rubber using polyacrylamide brush coatings	Flow rate of 0.025 mL s^−1^, corresponding to 10 s^−1^	4 h	Silicone wafers Silicone rubber Polyacrylamide brushes	*Staphylococcus aureus* *Streptococcus salivarius* *Escherichia coli* *Candida albicans*	A high reduction (52–92%) in microbial adhesion to the polyacrylamide brushes was observed compared to untreated silicon surfaces. The polymer brush did not cause surface deterioration and discouraged microbial adhesion, even after 1-month of exposure to physiological fluids.	[131,132]
	Implanted medical devices	Adhesion	Study of adhesion of bacterial and yeast strains to a poly(ethylene oxide) brush covalently attached to the glass	Flow rate of 0.025 mL s^−1^, corresponding to 10 s^−1^	4 h	Glass Poly(ethylene oxide) brushes on glass	*Staphylococcus epidermidis* *Staphylococcus aureus* *Streptococcus salivarius* *Escherichia coli* *Pseudomonas aeruginosa* *Candida albicans* *Candida tropicalis*	The poly(ethylene oxide) brush yielded more than 98% reduction in bacterial adhesion, although for the more hydrophobic *P. aeruginosa* a smaller reduction was observed. For yeast species, adhesion suppression was less effective than for the bacteria.	[133]
			Evaluation of the role of surface free energy on bacterial adhesion to plasma-modified films	50 and 200 s^−1^	2.5 h	Polyethylene terephthalate Plasma treated polyethylene terephthalate	*Staphylococcus epidermidis*	Plasma treatments reduced bacterial adhesion, in comparison to the untreated polymer. The ageing effect and the subsequent decrease in the surface free energy seemed to favor bacterial adhesion and aggregation. The increase in the shear rate restricted the predictability of the thermodynamic models.	[134]
		Adhesion and biofilm formation	Evaluation of the effectiveness of different formulations of a biomedical-grade polyetherurethane at inhibiting bacterial colonization under flow conditions	2.03 Pa	Adhesion: 2, 4 and 6 h Biofilm: 8, 20 and 24 h	Polyetherurethane Polyetherurethane with triglyme Polyetherurethane with poly(butyl methyacrylate) barrier membrane releasing ciprofloxacin	*Pseudomonas aeruginosa*	The rate of adherent cell accumulation was zero for the polyetherurethane with a poly(butyl methyacrylate) barrier membrane releasing ciprofloxacin.	[135]
	Surgical, catheters, and haemodialysis devices	Adhesion	Evaluation of the adhesion behavior of bacterial strains to hydrophilic and hydrophobic surfaces using theoretical predictions	Flow rate of 0.025 mL s^−1^, corresponding to 6 s^−1^	2 h	Glass Indium tin oxide-coated glass	*Pseudomonas stutzeri* *Staphylococcus epidermidis*	*P. stutzeri* has a much better adhesion rate than *S. epidermidis* for both material surfaces. Both bacterial strains adhered better to the hydrophobic indium tin oxide-coated glass than to the hydrophilic glass.	[136]
	Orthopedic implants	Adhesion	Study the bacterial adhesion to polymers that show promise as orthopedic materials	Flow rate of 1 mL min^−1^, corresponding to a shear rate of 1.9 s^−1^	1 h	Poly(orthoester) Poly(L-lactic acid) Poly(ether ether ketone) Polysulfone Polyethylene	*Staphylococcus epidermidis* *Pseudomonas aeruginosa* *Escherichia coli*	Tryptic soy broth decreased adhesion to polymers, when compared to phosphate-buffered saline. The estimated values of the free energy of adhesion correlated with the amount of adherent *P. aeruginosa.* There was 50% more adhesion of *E. coli* and *P. aeruginosa* on poly(orthoester) and poly(L-lactic acid) pre-exposed to hyaluronic acid. *P. aeruginosa* was the most adherent strain, while *S. epidermidis* was the least adherent strain.	[137]
	Urinary devices	Adhesion	Examination of the ability of probiotic strains to displace adhering cells from hydrophobic and hydrophilic substrata	15 s^−1^	4.5 h	Glass Fluorinated ethylene propylene	*Enterococcus faecalis*	*Ent. faecalis* was displaced by lactobacilli (31%) and streptococci (74%) from fluorinated ethylene propylene in buffer, and that displacement by lactobacilli was even more effective on glass in urine (54%). The passage of an air–liquid interface impacted adhesion, especially when the surface had been challenged with lactobacilli (up to 100%) or streptococci (up to 94%).	[138]
			Potential of biosurfactant layer to inhibit adhesion of uropathogens	Flow rate of 0.034 mL s^−1^, corresponding to 15 s^−1^	4 h	Glass Silicone rubber coated with different concentrations of a biosurfactant	*Enterococcus faecalis*	Biosurfactant layers inhibited the initial deposition rates (> 30%) and adhesion numbers (≈ 70–100%) in a dose-related way.For urine experiments, biosurfactant coatings caused higher adhesion reductions.	[122]
			Effect of supplementation on human urine and uropathogen adhesion	Flow rate of 0.034 mL s^−1^, corresponding to 15 s^−1^	4 h	Silicone rubber	*Escherichia coli* *Enterococcus faecalis* *Staphylococcus epidermidis* *Pseudomonas aeruginosa* *Candida albicans*	Cranberry and ascorbic acid supplementation can provide a degree of protection against adhesion and colonization of biomaterials by some uropathogens.	[139]
			Effect of combined surface chemistry and topography on bacterial adhesion	Flow rates of 2 and 4 mL s^−1^, corresponding to 0.010 and 0.024 Pa	0.5 h	Smooth polydimethylsiloxane Smooth polydimethylsiloxane with peptide coating Micropatterned polydimethylsiloxane Micropatterned polydimethylsiloxane with peptide coating	*Escherichia coli*	The highest adhesion was obtained on the smooth polydimethylsiloxane, whereas the micropatterned polydimethylsiloxane coated with peptide totally inhibited adhesion. The peptide addition to the smooth surface reduced the adhesion by 43–58%, while the micropatterned surface reduced the adhesion by 99%.	[21]
		Biofilm formation	Impact of temperature and surface on the biofilm-forming capacity of uropathogens	Flow rate of 4 × 10^−3^ mL s^−1^, corresponding to 33 s^−1^	20–24 h	Silicone Silicone coated with plasma polymerized vinylpyrrolidone	*Escherichia coli*	Temperature had a considerable influence upon the adhesion and biofilm-forming capacity of some of the isolates, and the influence of surface chemistry also depended on the temperature.	[140]
			Effect of applying different current densities to platinum electrodes as a possible catheter coating material	Flow rate of 3333 mL s^−1^, corresponding to 200 s^−1^	6 days	Platinum electrodes	*Proteus mirabilis*	By applying alternating microcurrent densities, a self-regenerative surface is produced, which removed the conditioning film and reduced bacterial adherence, growth, and survival.	[141]
		Biofilm formation and treatment	Potential of using a polymer brush on the prevention of biofilm formation and susceptibility	Flow rate of 2 mL s^−1^, corresponding to 15 s^−1^	Biofilm: 24 h Treatment: 8 h	Glass Polydimethylsiloxane Poly[oligo(ethyleneglycol) methyl ether methacrylate] brush	*Escherichia coli*	The polymer brush reduced the surface area and the number of total adhered cells by 57%. The antibiotic treatment potentiated cell death and removal (88%). The polymer brush has the potential to prevent biofilm growth and in eradicating biofilms developed in urinary devices.	[59]
			Effect of using a polymer brush on biofilm cell composition and architecture	Flow rate of 2 mL s^−1^, corresponding to 15 s^−1^	Biofilm: 24 h Treatment: 8 h	Glass Polydimethylsiloxane Poly[N-(2-hydroxypropyl) methacrylamide] brush	*Escherichia coli*	Initial adhesion and surface coverage decreased on the polymer brush. Viable but nonculturable cells were completely removed from the brush. The polymer brush may reduce biofilm growth and antibiotic resistance in urinary catheters.	[58]
**96-well microplates**	Biomedical scenarios	Biofilm formation	Evaluation of the combined effects of shear forces and nutrient levels on biofilm formation and definition of the operational conditions to be used to simulate relevant biomedical scenarios	Orbital shaking with 25 and 50 mm diameter incubators at 150 rpm (average shear rate of 23 and 46 s^−1^)	60 h	Polystyrene	*Escherichia coli*	Higher glucose concentrations enhanced *E. coli* adhesion in the first 24 h, but variations in peptone and yeast extract concentrations had no significant impact on biofilm formation. Numerical simulations indicate that 96-well microplates can be used to simulate a variety of biomedical scenarios if the operating conditions are carefully set.	[68]
**Microfluidic device**	General medical devices	Adhesion	Development of a fabrication method to produce a microfluidic device to test cell adhesion	0.01–1 Pa	0.5 h	Polyamide Polydimethylsiloxane Polyethylene oxide Poly(L-lactic acid) Polystyrene	*Escherichia coli*	Bacterial adhesion increased linearly over time. The evaluation performed with polydimethylsiloxane for shear stresses between 0.02 and 1 Pa showed that the lowered surface (inherent weakness of the fabrication method) did not influence adhesion.	[113]
			Study the initial cell adhesion dependence on local wall shear stress in a microchannel with intercalate zones of constrictions and expansions	0.2–10 Pa	0.5 h	Glass	*Escherichia coli*	Bacterial adhesion increased in locations with a sudden increase in shear stress.	[142]
			Examination of the role of surface properties on bacterial adhesion	0.002–0.042 Pa	n.d.	Smooth silicone Patterned silicone	*Escherichia coli*	Cell attachment was observed to be strongly dependent upon the topographical features. The highest attachment density was observed on smooth surfaces.	[114]
		Biofilm formation	Comparison of the biofilm-forming capacities of various Methicillin-resistant *Staphylococcus aureus* clones	0.05 Pa	18 h	Glass	Methicillin-resistant *Staphylococcus aureus*	From tested isolates, 51% successfully formed biofilms under shear flow. Differences in biofilm formation might also be due to the different adherent surfaces.	[143]
			Study of biofilm formation and host–pathogen interactions	0.05–1 Pa	24 h	Glass Eukaryotic cells (HRT-18)	*Escherichia coli*	Biofilm formation on glass was observed for most strains in M9 medium at 30 °C. HRT-18 cell monolayers enhanced *E. coli* binding and biofilm formation.	[144]
	Implanted medical device	Biofilm formation	Investigation of how environmental factors, such as surface geometry and chemistry, as well as fluid flow, affect biofilm development	0.02–1 Pa	16 h	Uncoated and human blood plasma-coated channels	*Staphylococcus aureus*	The flow was the major contributor to the shape of biofilm structures, whereas bacterial motility was less significant.	[115]
	Mammary environment	Biofilm formation	Evaluation of the effect of coagulase-negative staphylococci isolates with a weak- biofilm phenotype	0.05 Pa	24 h	Glass	Coagulase-negative staphylococci	Coagulase-negative staphylococci with a weak biofilm phenotype did not inhibit the growth of isolates with a strong-biofilm phenotype.	[145]
	Intravascular catheter	Biofilm formation	Investigation of flow as an environmental signal for biofilm formation	Flow rates of 1–10 mL h^−1^, corresponding to 0.065–1.14 Pa	24 h	Channels treated with octyl(tri-ethoxy)silane	*Staphylococcus epidermidis*	Fluid shear alone induced the formation of polysaccharide intracellular adhesin-positive biofilms and influenced the biofilm structure.	[116]
	Urinary devices	Adhesion	Development of microfluidic-based devices replicating the urodynamic field within different configurations of an occluded and stented ureter	Up to 0.175 Pa	1 h	Polydimethylsiloxane	*Pseudomonas fluorescens*	The unobstructed device showed no bacterial attachment, including in regions of low shear stress (<0.04 Pa). For the obstructed devices, the cavity region, and the nearby proximal side-hole (shear stresses of 0.131–0.175 Pa) exhibited greater levels of bacterial attachment (18%) compared to other regions of the model.	[63]

n.d.—not disclosed; D. tsuruhatensis—Delftia tsuruhatensis, Ent. faecalis—Enterococcus faecalis, E. coli—Escherichia coli, P. aeruginosa—Pseudomonas aeruginosa, P. stutzeri—Pseudomonas stutzeri, S. epidermidis—Staphylococcus epidermidis.

## 5. Current Challenges and Future Directions of Biofilm Platforms Research

Although biofilms are a recognized problem for the environment, industry, and medicine, and act as a possible reservoir of pathogens, there is a lack of reliable standard procedures to evaluate the efficacy of methods for biofilm prevention and removal. Consequently, it is very difficult to compare data obtained in different laboratories. As discussed before, laboratory reactors are available for growing biofilms that are more representative of a clinical situation [37,146] and industrial environment [147]. Although commercially available reactors with standardized protocols exist (e.g., ASTM Method E2871-13 and 2562-12 for the CDC biofilm reactor [148]), they are usually expensive and, thus, not accessible to all biofilm researchers, besides that the operation of these reactors has specific limitations. For instance, some of them cannot be used to test different surface materials, have reduced sampling areas, require specialized labor for operation, and the fluid dynamics are rarely well-characterized. While factors such as the temperature, microbial composition, and carbon source may be similar across different protocols and biofilm platforms, the fluid dynamics, namely the shear stress and shear rate, are a defining feature of a particular reactor operation. Whether the researchers are using a commercial or custom-made biofilm setup, computational simulations of hydrodynamics are extremely valuable, as they enable a more informed decision about whether the flow behavior in that specific biofilm reactor is suitable for their research.

Nevertheless, not all interactions between early adhered cells or established biofilms and fluid flow phases (gas and/or liquid) are considered when using the CFD technique. Almost all the flows in the described biofilm reactors deal with multiphase (gas–liquid, solid–liquid, and gas–liquid–solid), but some simplifications are introduced to reduce the model complexity [78,149]. For example, the aeration of flow cell systems is often not taken into account in the CFD study [43]. Furthermore, one must bear in mind that numerical simulations are mostly performed for clean and perfectly smooth surfaces. However, as biofilms grow or different coupon materials are used, the surface properties (such as roughness and hydrophobicity) should be considered for their impact on the wall shear stress. Therefore, there is still a great challenge in the integration of physical and biological processes in biofilm reactors.

Small flow chambers and microfluidic platforms are promising for screening new possible antibiofilm approaches. They need smaller volumes of media and reagents to run continuous biofilm experiments, when compared to the Robbins device and rotating biofilm reactors, enabling high-throughput assays. Additionally, the Bioflux [150] and other microfluidic devices [151,152] are dynamic systems with significant potential for monitoring heterogeneity in the biofilm microenvironment [153]. This can be achieved with specific stains and examination by confocal microscopy. However, direct biofilm observation might not be feasible, specific stains/probes may not be available (for nutrients or metabolites), or the time scale may be too slow. Introducing sensing techniques, such as microsensors or electrochemical probes in microfluidic chips, is an important development for online biofilm detection and microenvironment analysis [153].

## 6. Conclusions

Studying microbial adhesion and biofilm growth is crucial for understanding the physiology of sessile organisms and forming the basis for the development of novel antimicrobial materials. Fluid hydrodynamics is one of the most important factors affecting cell adhesion, as well as biofilm structure and behavior. Therefore, to simulate the relevant biofilms of different fields (environment, industry, and medicine) in the laboratory, it is of utmost importance to select an adequate biofilm platform and be able to operate it at hydrodynamic conditions that are as close as possible to those encountered in a real scenario.

## Figures and Tables

**Table 1:** Main advantages and limitations of standard platforms for in vitro biofilm studies [36,38].

Platform	Advantages	Disadvantages
Modified Robbins device	Large amount of biomass is produced	Complex setup
	High/moderate throughput	Entry effects
	Allows periodical sampling	Sampling can affect the biofilm
	Can be run for very long periods without intervention	Limited in situ biofilm visualization
	Large dynamic range	Biofilm destruction for most quantitative analysis
Flow chamber	Optimized for online in situ microscopy	Low throughput
	Allows direct and nondestructive observation of biofilm development	Inability to study adhesion to nontransparent surfaces
		Complex setup
Rotating biofilm devices	Possibility to study different materials in the same run	The flow pattern changes in the boundaries of the coupons
	Shear stress and feed flow rate can be set independently	Lack of sampling surface area
	Easy to control the operational conditions	Complex setup
		Expensive
Microplates	High-throughput analysis	Direct observation under the microscope can be difficult
	Simple to run	Batch system
	Needs small space	Loosely attached biofilm may not be correctly quantified
	Inexpensive	Operator dependent
Microfluidic devices	Noninvasive technique	Requires special equipment for manufacturing and running systems
	Allows real-time visualization of biofilm development	Clogging can occur due to small dimensions
	Requires small volumes	Laborious operation
	Can be custom made for specific purposes	Air bubbles may be an issue
	Rapid and precise analysis	Viscosity effects may arise
	Compatible with single-cell analysis	

**Table 3:** Environmental studies performed on different biofilm platforms to evaluate the initial adhesion, biofilm formation, and treatment under defined shear conditions.

**Platform**	**Field**	**Biofilm Stage**	**Study Aim**	**Hydrodynamics**	**Assay Time**	**Surface** **Material**	**Organisms**	**Concluding Remarks**	**References**
**Modified Robbins device**	Drinking- water distribution systems	Biofilm formation	Investigate the combined impact of flow hydrodynamics and pipe material	0.13 and 0.24 Pa	100 days	Polyvinyl chloride Polypropylene Structured wall high-density polyethylene Solid wall high-density polyethylene	Natural flora present in drinking water	The biomass amount was greater for the biofilms formed at lower shear stress. The opportunistic pathogens have limited ability to propagate within biofilms under high shear conditions without protection (surface roughness).	[79]
	Water treatment	Biofilm formation	Evaluate the application of non-biocide release coatings as coated filters for biofouling prevention	Flow rate of 300 L h^−1^, corresponding to an average shear stress of 0.25 Pa	2 days	Polyurethane coating Polyurethane coating with incorporated Econea Polyurethane coating with grafted Econea	*Enterococcus faecalis*	Biocidal polyurethane-based surfaces were less prone to biofilm formation, with an average reduction of 60%, compared to pristine polyurethane.	[81]
**Flow chamber**	Man-made equipment (heat exchangers, ship hulls, and pipelines)	Biofilm formation	Study the influence of surface energy components on the adhesion and removal of fouling	9.8 × 10^−4^, 4.6 × 10^−4^, and 2.1 × 10^−4^ Pa	10 days	316 L Stainless steel Ni–P–TiO_2_–polytetrafluoroethylene nanocomposite coatings	*Pseudomonas fluorescences* *Cobetia marina* *Vibrio alginolyticus*	Coatings with the lowest ratio between the Lifshitz van der Waals apolar component and the electron donor component had the lowest bacterial adhesion or the highest bacterial removal.	[85,86]
**Rotating cylinder reactor**	Drinking- water distribution systems	Biofilm formation and treatment	Effect of chemical and mechanical stresses on single and dual- species biofilm removal	Biofilm formation: 1 Pa Treatment: 1–23 Pa	7 days	Polyvinyl chloride	*Acinectobacter* *calcoaceticus* *Stenotrophomonas maltophilia*	Dual species biofilms were the most susceptible to chemical and mechanical removal. *Stenotrophomonas maltophilia* biofilms demonstrated high tolerance to chemical and mechanical stress.	[80]
		Biofilm formation	Action of copper materials on biofilm formation and control by chemical and mechanical stress	0.1 Pa	7 days	Stainless steel Copper alloys (100, 96, and 57%)	*Stenotrophomonas maltophilia*	Chemical, mechanical, and combined shocks were not effective in biofilm control. Copper surfaces were found to reduce the number of non-damaged cells.	[87]
**6-well microplates**	Drinking- water distribution systems	Adhesion and biofilm formation	Influence of shear stress, temperature, and inoculation concentration on water-stressed *Helicobacter pylori*	0, 60, and 120 rpm corresponding to 0, 0.138, and 0.317 Pa	2, 6, 12, 24, 48, 96, and 192 h	304 stainless steel Polypropylene	*Helicobacter pylori*	High shear stresses negatively influenced the adhesion to the substrata. However, the temperature and inoculation concentration appeared to not affect adhesion.	[88]
**12-well microplates**	Marine environment	Biofilm formation	Effect of surface hydrophobicity on biofilm development by a filamentous cyanobacterium	Orbital shaking with a 25 mm diameter incubator at 185 rpm (average shear stress of 0.07 Pa)	3 weeks	Glass Perspex	*Leptolyngbya mycoidea* LEGE 06118	Higher biofilm growth was observed on perspex, the most hydrophobic surface.	[89]
			Effect of different marine coatings on biofilm formation by microfoulers	Orbital shaking with a 25 mm diameter incubator at 185 rpm (average shear rate of 40 s^−1^)	7 weeks	Epoxy-coated glass Silicone hydrogel coating	*Cyanobium* sp. LEGE 10375 *Pseudoalteromonas tunicata* (marine bacterium)	Epoxy-coated surface was effective in inhibiting biofilm formation at the initial stages, while silicone coating showed high antibiofilm efficacy during maturation. Silicone coating was less prone to biofilm formation. The efficacy of silicone may be dependent on the organism, while the performance of epoxy-coated surface was strongly influenced by a combined effect of surface and microorganism.	[82]
			Effect of different materials on biofilm structure		7 weeks	Glass Perspex Polystyrene Epoxy-coated glass Silicone hydrogel coating	Synechocystis salina *LEGE 00041* *Cyanobium* sp. LEGE 06098 *Cyanobium* sp. *LEGE 10375*	Silicone coating was effective in inhibiting cyanobacterial biofilm formation. Cyanobacterial biofilms formed on silicone coating showed a lower percentage and size of empty spaces among all surfaces.	[70]
			Study the environmental compatibility of an innovative biocidal foul-release multifunctional coating		7 weeks	Polydimethylsiloxane Polydimethylsiloxane coating with grafted Econea	*Pseudoalteromonas tunicata*	Polydimethylsiloxane coating with grafted Econea was more effective in inhibiting biofilm formation than the bare polydimethylsiloxane (reductions of 77%, 60%, and 73% on biovolume, thickness, and substratum coverage, respectively). Long-lasting antifouling performances were observed in simulated and real scenarios.	[22]
			Effect of shear forces on biofilm development by filamentous cyanobacteria	Orbital shaking with a 25 mm diameter incubator at 40 rpm (average shear rate of 4 s^−1^) and 185 rpm (average shear rate of 40 s^−1^)	7 weeks	Glass Perspex	*Nodosilinea* sp. LEGE 06020 *Nodosilinea* sp. LEGE 06022 Unidentified filamentous *Synechococcales* LEGE 07185	Biofilm formation was higher under low shear conditions. The hydrodynamics was more effective on biofilm maturation than during initial cell adhesion. Different shear rates affected biofilm architecture.	[71]
			Effect of shear forces and surface hydrophobicity on biofilm development by coccoid cyanobacteria with different biofilm formation capacities		6 weeks	Glass Epoxy-coated glass	*Synechocystis salina* LEGE 00041 *Cyanobium sp.* LEGE 06097	Biofilms developed in both surfaces at lower shear conditions had a higher number of cells, wet weight, thickness, and chlorophyll *a* content. The impact of hydrodynamics was generally stronger than the impact of surface hydrophobicity. The antibiofilm performance of the polymeric coating was confirmed.	[90]
			Qualitative proteomic analyses of filamentous cyanobacterial biofilms formed under different shear rates		7 weeks	Glass Perspex	*Nodosilinea* sp. LEGE 06145 *Nodosilinea* sp. LEGE 0611	Biofilm formation was higher under low shear conditions. Biofilm development of *Nodosilinea* sp. LEGE 06145 was higher than LEGE 06119, but no significant differences were found between surfaces.	[91]
		Adhesion and biofilm formation	Potential of adhesion assays on the estimation of biofilm development behavior at different hydrodynamic conditions		Adhesion: 7.5 h Biofilm: 6 weeks	Glass Epoxy-coated glass	*Synechocystis salina* LEGE 00041 *Synechocystis salina* LEGE 06155 *Cyanobium* sp. LEGE 06097	For both adhesion and biofilm assays, the number of adhered cells was higher under low shear conditions. Higher biofilm development at 4 s^−1^ was confirmed by biofilm wet weight, thickness, and chlorophyll *a* content. Initial adhesion assays can be used to estimate marine biofilm development.	[92]
			Quantitative proteomic analyses of biofilms formed on different surfaces		7 weeks	Glass Perspex	Unidentified filamentous cyanobacterium LEGE 06007	After 7 weeks, high biofilm thickness was observed in biofilms formed at 4 s^−1^ on glass when compared to perspex. Differences in protein expression were more noticeable in biofilms formed under low shear conditions. Proteomic analysis revealed differentially expressed proteins between surfaces.	[93]

**Table 5:** Industrial studies performed on different biofilm platforms to evaluate the initial adhesion, as well as biofilm formation and treatment under defined shear conditions.

**Platform**	**Biofilm Stage**	**Study Aim**	**Hydrodynamics**	**Assay Time**	**Surface** **Material**	**Organisms**	**Concluding Remarks**	**References**
**Modified Robbins device**	Biofilm formation	Effect of flow rate/shear stress variation on mass transfer and biofilm development in a flow cell that mimics industrial piping	Flow rates of 374 and 242 L h^−1^, corresponding to wall shear stresses between 0.183 and 0.511 Pa	9 days	Polyvinyl chloride	*Escherichia coli*	Biofilm formation was favored at the lowest flow rate. Shear stress effects were more important than mass transfer limitations. This flow cell system generates wall shear stresses that are similar to those found in some industrial settings.	[111,112]
	Biofilm formation and treatment	Evaluation of a modified diamond-like carbon surface for biofouling mitigation in critical process areas	Flow rate of 300 L h^−1^, corresponding to an average shear stress of 0.25 Pa	Biofilm formation: 5 days Treatment: 6, 18, and 24 h	316 L Stainless steel SICON^®^	*Escherichia coli* Natural flora present in the water from an industrial salad washing line	Biofilm formation was reduced on SICON^®^ (1–2 Log). Biofilm cleaning with chlorine was more efficient when SICON^®^ was used (3.5-Log reduction and 15% removal). Industries with cleaning frequencies up to 6 h may benefit from the use of SICON^®^.	[41]
	Biofilm formation and treatment	Evaluation of SICAN for biofouling mitigation in the food industry		Biofilm formation: 5 days Treatment: 6, 18 and 24 h	316 L Stainless Steel SICAN	*Escherichia coli* Natural flora present in the water from an industrial salad washing line	Biofilm formation on SICAN and stainless steel were similar. Processes with cleaning intervals of about 6 h could potentially use SICAN surfaces on critical areas.	[24]
**Flow** **chamber**	**Adhesion**	Effect of strain, shear stress, surface soiling, and growth conditions on *Listeria monocytogenes* adhesion	Flow rates of 0.76 and 10.9 mL min^−1^, corresponding to wall shear stresses of 0.0505 and 0.7620 Pa	30 min	Glass Polyvinyl chloride Glass coated with beef extract, casein, and milk	*Listeria monocytogenes*	Strain differences influenced the initial adhesion rate to all the surfaces at both low and high shear stress. There was a significant effect of the surfaces on the adhesion ability of almost all strains. The initial adhesion rate decreased at high shear stress for most strains.	[97]
		Effect of flow direction and flow rate on the initial adhesion of *Listeria monocytogenes* strains	Flow rates of 0.75 and 8.40 mL min^−1^, corresponding to wall shear stresses of 0.10 and 1.20 Pa	15 min	Fine polished stainless steel	*Listeria monocytogenes*	Initial adhesion rates were influenced by flow rate and strain specificity. The flow direction, in relation to the orientation of surface features, could be disregarded.	[98]
	Biofilm formation	Effect of surface conditioning on adhesion and biofilm formation under conditions that are prevalent in the food industry	Flow rate of 11 mL s^−1^, corresponding to an average shear stress of 0.07 Pa	24 h	Polystyrene Polystyrene conditioned with cell extracts and cell wall components	*Escherichia coli*	Under flow conditions, all conditioning films reduced biofilm formation, except mannose. Surface conditioning affected the amount and clustering of bacteria on surfaces.	[8,77]
**Rotating cylinder reactor**	Biofilm formation and treatment	Effect of shear stress on the formation and removal of biofilms	0.02, 0.12, and 0.17 Pa	Biofilm formation: 7 days Treatment: 0.5 h	AISI 316 Stainless steel	*Bacillus cereus*	Biofilm density increased with the shear stress, while the thickness decreased. The biocide treatment promoted the higher removal of biofilms formed under higher shear stress. Biofilms formed under higher shear stress were more resistant to the mechanical and combined biocide and mechanical treatments.	[95]
**Rotating disk reactor**	Biofilm formation	Effect of shear stress on biofilm formation	Rotational speeds of 350 and 800 rpm, corresponding to shear stresses between 0 and 91 Pa	4 days	AISI 304 2B food grade stainless steel	*Candida krusei*	The early development of a biofilm (24 h) was unaffected by shear stress. In a mature biofilm, shear stress determined the disposition of biofilm cells onto the surface. Biofilms formed under higher shear stress differ in their arrangement, as compared with those formed under lower shear conditions.	[96]
		Assessment of the colonization of biofilms by free-living amoebae	Shear rates between 31,000 and 85,000 s^−1^, representative of cooling circuits	10 days	Stainless steel	Freshwater containing free-living amoebae and bacteria	Free-living amoebae were able to establish in biofilms under shear rate as high as 85,000 s^−1^. The developed reactor seems to be ideal for studying the effects of high shear stress on surface colonization by microorganisms.	[94]
**96-well microplates**	Adhesion	Effect of surface conditioning on adhesion and biofilm formation under conditions that are prevalent in the food industry	Orbital shaking with a 50 mm diameter incubator at 150 rpm (average shear stress of 0.07 Pa)	1 h	Polystyrene Polystyrene conditioned with cell extracts and cell wall components	*Escherichia coli*	Total cell extract, cytoplasm with cellular debris, myristic, and palmitic acid decreased initial adhesion. Adhesion increased when periplasmic extract was used. Adhesion was dependent on the conditioning film concentration.	[8,77]
**6-well microplates**	Adhesion	Evaluation of the antiadhesive activity of SICON^®^	Orbital shaking with a 25 mm diameter incubator (average shear stress of 0.25 Pa)	0.5, 2, and 6 h	316 L Stainless steel SICON^®^	*Escherichia coli* Natural flora present in the water from an industrial salad washing line	Bacterial adhesion on SICON^®^ and stainless steel were similar.	[41]
	Adhesion	Evaluation of the antiadhesive activity of SICAN	Orbital shaking with a 25 mm diameter incubator (average shear stress of 0.25 Pa)	0.5, 2, and 6 h	316 L Stainless steel SICAN	*Escherichia coli* Natural flora present in the water from an industrial salad washing line	Adhesion on SICAN and stainless steel were similar. *Escherichia coli* and the flora from industrial water had similar adhesion behaviour.	[24]
	Adhesion and biofilm formation	Assessment of the impact of material properties, nutrient load, and shear stress on biofouling in food industries	Static and orbital shaking with a 25 mm diameter incubator at 115 rpm (average shear stress of 0.27 Pa)	Adhesion: 0.5 h Biofilm: 6 h	Glass Copper Stainless steel	*Escherichia coli*	Surface material was the most important factor in adhesion and biofilm formation. Adhesion and biofilm formation were correlated with surface hydrophobicity. The effect of surface properties was dependent on the nutrient load and shear stress. Initial adhesion performance may be a good predictor for biofilm formation.	[73]
